# Are we ready? What is missing and what is needed? A regulator’s perspective

**DOI:** 10.1186/1750-1172-9-S1-O25

**Published:** 2014-11-11

**Authors:** Claudia Bernardini, Luisa AA Muscolo, Paolo D Siviero, Simona Montilla, Luca Pani

**Affiliations:** 1 Italian Medicines Agency (AIFA) Rome, Italy

## Background

The lack of clinical data for orphan drugs and the development of “precision medicines” or advanced therapies have required a new regulatory approach to guarantee a marketing authorization for new and innovative medicines.

What is actually changing in the scenario is the way Agencies are managing the regulatory procedures to ensure a rapid access to treatment for patients with unmet medical needs.

In the last few years EMA and National Agencies have provided a lot of scientific advices (SA) to companies and applicants about orphan designation, CTs design, marketing authorisation procedures and HTA evaluations.

Currently the global scenario is already changing as we are issuing licenses for marketing authorization under exceptional circumstances and conditional marketing authorizations. The legislative framework is also changing with the new Pharmacovigilance Directive and the new Regulation on Clinical Trials entering into force progressively by 2015.

## Materials and methods

The new Pharmacovigilance Legislation supports a Progressive Patient Access Scheme (PPAS, or “Adaptive Licensing”) approach by expanding EMA authority to request mandatorily to Applicants a post-marketing evaluation of effectiveness in addition to safety.

The basic principles of PPAS approaches are facilitating early access to an early medicine approval, by acknowledging and managing uncertainty about favourable and unfavourable effects. The result will be achieved through the conduction of optimised RCTs, the monitoring of risk-based SA and the harmonisation of RCTs platforms.

Lots of effort is needed by everyone through a new level of cooperation, in particular regulators, companies and the CROs need to go down to patient level in order to evaluate how the trial is proceeding and by avoiding complex policies.

Moreover, patients have to accept a certain level of uncertainty as the clinical studies are not completed and Clinical Trials investigators need a continuous talking to patients during the drug development to ensure the higher level of achievable safety.

## Results and conclusions

A successful PPAS pathway depends on the willingness of patients, regulators, health-care providers and payers to accept a greater level of uncertainty using the new medicine in the expectation of an improved benefit risk profile.

Furthermore, PPAS approach could facilitate a more open and timely strong scientific dialogue and significant cooperation among stakeholders and a time reduction to full market approval resulting into a possible impact reduction on the overall cost of development.

